# Semi-empirical study of *ortho*-cresol photo degradation in manganese-doped zinc oxide nanoparticles suspensions

**DOI:** 10.1186/1752-153X-6-88

**Published:** 2012-08-21

**Authors:** Yadollah Abdollahi, Azmi Zakaria, Abdul Halim Abdullah, Hamid Reza Fard Masoumi, Hossein Jahangirian, Kamyar Shameli, Majid Rezayi, Santo Banerjee, Tahereh Abdollahi

**Affiliations:** 1 Material Synthesis and Characterization Laboratory, Institute of Advanced Technology, Universiti Putra Malaysia, 43400, Serdang, Selangor, Malaysia; 2 Department of Chemistry, Faculty of Science, Universiti Putra Malaysia, 43400, Serdang, Selangor, Malaysia; 3 Laboratory of Cryptography, Analysis and Structure, Institute for Mathematical Research, Universiti Putra Malaysia, and Department of Complexity and Network Dynamics International Science Association, Ankara, Turkey; 4 School of Chemical Sciences and Food Technology, Faculty of Science and Technology, Universiti Kebangsaan Malaysia, 43600, Bangi, Selangor D.E, Malaysia

**Keywords:** Photo degradation, Mn-doped ZnO nanoparticles, Optimization, Modeling, RSM, Simulation, ANOVA, Semi-empirical, Photo catalyst

## Abstract

The optimization processes of photo degradation are complicated and expensive when it is performed with traditional methods such as one variable at a time. In this research, the condition of *ortho*-cresol (*o*-cresol) photo degradation was optimized by using a semi empirical method. First of all, the experiments were designed with four effective factors including irradiation time, pH, photo catalyst’s amount, *o*-cresol concentration and photo degradation % as response by response surface methodology (RSM). The RSM used central composite design (CCD) method consists of 30 runs to obtain the actual responses. The actual responses were fitted with the second order algebraic polynomial equation to select a model (suggested model). The suggested model was validated by a few numbers of excellent statistical evidences in analysis of variance (ANOVA). The used evidences include high F-value (143.12), very low P-value (<0.0001), non-significant lack of fit, the determination coefficient (R^2^ = 0.99) and the adequate precision (47.067). To visualize the optimum, the validated model simulated the condition of variables and response (photo degradation %) be using a few number of three dimensional plots (3D). To confirm the model, the optimums were performed in laboratory. The results of performed experiments were quite close to the predicted values. In conclusion, the study indicated that the model is successful to simulate the optimum condition of *o*-cresol photo degradation under visible-light irradiation by manganese doped ZnO nanoparticles.

## Background

Advanced oxidation processes (AOPs) are physicochemical procedures, which are designed to remove environmental organic and inorganic pollutions. Photo catalysis, the current interest of AOPs, is applied for decontamination of environmental organic pollutions [1-4]. The photo catalysis, under suitable light illumination, produces hydroxyl radical (^●^OH) and hole (h^+^) which are powerful and non-selective oxidants to degrade a variety of organic compounds [5-7]. Zinc oxide (ZnO) with great advantage in absorption a larger fractions of the solar spectrum, is one of the photo catalyst that removed several environmental contaminants under visible-light irradiation [8-11]. To enhance the visible-photo activity of ZnO, doping the transition metals such as manganese (Mn) has been prepared [12]. In the most cases, the evaluation of the photo degradation was carried out by a traditional method (one variable at a time), which is not only costly and time consuming but also often leads to misinterpretation of Experimental results [13-19]. Since the photo degradation is dependent on several effective parameters such as irradiation time, pH value, photo catalyst loading and substrate concentration [11,20], it needs to preform too many experiments for finding the optimum condition. While the managing of these experiments is difficult, the real world of photo degradation’s experiments is very expensive because of chemicals, instruments, time of the researchers and etc. Therefore, prediction of the optimal condition seems necessary. Recently the response surface methodology (RSM) was used as an efficient technique for these purposes (simulation the condition) [21-26]. In the methodology, central composite design (CCD) was used for experimental-design and fitting the performed (actual) results with a polynomial equation in vicinity region of the optimum condition to make a model [27]. The model relates the responses (sometimes yield) and the variables of the photo degradation process [28]. However, no study has been conducted on application of the RSM that have been reported on the photo degradation of *ortho*-cresol (*o*-cresol) by manganese-doped zinc oxide (Mn-doped ZnO) nanoparticles as photo catalyst. In this work, the optimum condition was simulated by the RSM and then visualized by 3D plots in vicinity region of reported optimum condition [29]. The predicted optimum of the responses and the variables were confirmed by the actual responses of the laboratories experiments.

## Experiment

### Empirical methodology

*O*-cresol (99%, Fluka), NaOH (99% Merck), H_2_SO_4_ (95%-97%) are of reagent grade, obtained from Merck which used without further purification. Mn-doped ZnO nanoparticles (photo catalyst) were synthesized according to procedures that is previously described [12]. Photocatalic degradation of *o*-cresol was performed in a designed batch photo reactor with Philips lamp (23 watt) as visible light source [30]. Throughout the study, a desired concentration of *o*-cresol solution was mixed with an appropriate amount of photo catalyst in the photo reactor. At specific time intervals, aliquot samples were withdrawn from the bulk solution and filtered through 0.2 μm polytetrafluoro ethylene (PTFE) filters. The concentration of *o*-cresol was measured using UV-visible spectrophotometer (shimadzu, uv-1650pc).

### Statistical methods

To find the optimum conditions of the photo degradation, the experiments were designed by RSM and CCD (Table 1 the design is in code). The design with four effective variables (Table 2) was run by the design-expert version 8.0.7.1. The plan was included 16 factor points (2^n^), 8 axial points (2n), and 6 center points (replications) the total numbers of performed runs were 30 experiments. The designed actual responses were fitted to the linear, 2FI, quadratic and cubic models by CCD. The fitting was based on a second order polynomial model (Eq. 1) by a multiple regression analysis [27],

(1) $$Y=\beta_{0}+\sum_{i=1}^{4}\beta_{i}\chi_{i}+\sum_{i=1}^{4}\beta_{ii}\chi_{i}^{2}\sum_{i=1}^{3}\sum_{j=i+1}^{4}\beta_{ij}\chi_{i}\chi_{j}+\varepsilon$$

**Table 1:** **Experimental-Design of *o*-cresol photo degradation**

**Std**	**Run**	**X**_**1**_	***X***_**2**_	**X**_**3**_	**X**_**4**_	**Y (%)**
12	1	1	1	-1	1	
3	2	-1	1	-1	-1	
28	3	0	0	0	0	
11	4	-1	1	-1	1	
18	5	2	0	0	0	
29	6	0	0	0	0	
19	7	0	-2	0	0	
6	8	1	-1	1	-1	
21	9	0	0	-2	0	
13	10	-1	-1	1	1	
24	11	0	0	0	2	
4	12	1	1	-1	-1	
25	13	0	0	0	0	
23	14	0	0	0	-2	
14	15	1	-1	1	1	
5	16	-1	-1	1	-1	
8	17	1	1	1	-1	
22	18	0	0	2	0	
9	19	-1	-1	-1	1	
2	20	1	-1	-1	-1	
20	21	0	2	0	0	
15	22	-1	1	1	1	
17	23	-2	0	0	0	
7	24	-1	1	1	-1	
27	25	0	0	0	0	
1	26	-1	-1	-1	-1	
26	27	0	0	0	0	
30	28	0	0	0	0	
10	29	1	-1	-1	1	
16	30	1	1	1	1	

**Table 2:** Independent variables and their levels employed in the central composite design

**Variables**		**Units**	**Level of variables**	
			**Low**	**High**
X_1_	Irradiation time	minute	120	360
*X*_2_	pH	-	6.6	9.8
X_3_	Photo catalyst amount	g/L	0.5	2.5
X_4_	Concentration of *o*-cresol	mg/L	15	55

where *Y (*photo degradation %*)* represents the response variable, *β*_*0*_ is the constant term, *β*_*i*_ represents the coefficients of the linear parameters, x_i_ represents the variables, *β*_*ii*_ represents the coefficients of the quadratic parameter, *β*_*ij*_ represents the coefficients of the interaction parameters and ε is the residual associated to the experiments. The significance and adequacy of the model was determined by a few numbers of statistical evidences that appear in analysis of variance (ANOVA) as output of the CCD method. These evidences include Fisher variation ratio (F-value), probability value (P-value), Lack of Fit, coefficient of determination R-squared (R_d_^2^), adjusted R-squared (R_Adj_^2^), predicted R-squared (R_Pred_^2^) and adequate precision of Predicted Residual Error of Sum of Squares (PRESS). Most of these parameters are clearly defined in the experimental design texts. PRESS is a signal-to-noise ratio, which compares the range of the predicted values at the design points to the average prediction error. The ratios greater than 4 indicate adequate model discrimination [30]. R_Adj_^2^ and the R_Pred_^2^ are the measurement of the amount of variation around the mean and the new explained data, respectively. The very significant is the Fisher test where P-value is compared with F-value. F-value is a statistically valid measure of how well the factors described the variation in the data about its meaning while P-value represents the degree of significance of each variable. Mathematically, F-value is given by the ratio of mean square due to model variation by that due to error variance (Eq. 2). The high value of F-value indicates significance and adequacy of the model,

(2) $$F-\text{value}\;={S_{r}}^{2}/{S_{e}}^{2}$$

where S_r_^2^ and S_e_^2^are mean of the model and residuals square respectively, obtained by dividing the sum of squares of each of the two sources of variation, the model, and the error variance, by the respective degrees of freedom (DF) [27]. For a variable having a P*-*value smaller than 0.05, response would be influenced at a confidence level of 0.95.

## Analysis of the results

### Satisfactory adjustment of the model

Table 3 shows the ANOVA of the quadratic model for the photo degradation. A high F-value (F_model_ = 143.12) was obtained while there is only 0.01% chance of occurrence of noise, indicating substantial significance of the model. The Prob > F (<0.0001) of model is much smaller than 0.05 which indicates the most terms of the model including (X_1_, *X*_2_, X_3_, X_4_, X_2_X_3_, X_2_X_4_, X_2_^2^, X_3_^2^, X_4_^2^) are significant (reckoning that the values greater than 0.1 are indication of the model terms are not significant). Pure errors such as experimental errors are minimal as the Lack of Fit is not significant ((=1.72).

**Table 3:** **Analysis of the variance for photo catalytic degradation of*o*-cresol parameters**

**Source**	**Sum of squares**	**Degree of freedom**	**Mean square**	**F Value**	**Prob > F**
Model	12227.40	14	873.39	143.12	< 0.0001
X_1_	3658.07	1	3658.07	599.43	< 0.0001
*X*_2_	184.26	1	184.26	30.19	< 0.0001
X_3_	497.77	1	497.77	81.57	< 0.0001
X_4_	5701.08	1	5701.08	934.20	< 0.0001
X_1_^2^	14.25	1	14.25	2.34	0.1473
X_2_^2^	10.40	1	10.40	1.70	0.2114
X_3_^2^	0.53	1	0.53	0.09	0.7732
X_4_^2^	271.43	1	271.43	44.48	< 0.0001
X_1_*X*_2_	43.89	1	43.89	7.19	0.0171
X_1_ X_3_	0.076	1	0.076	0.01	0.9128
X_1_ X_4_	9.98	1	9.98	1.63	0.2205
*X*_2_ X_3_	491.79	1	491.79	80.59	< 0.0001
*X*_2_ X_4_	1040.58	1	1040.58	170.51	< 0.0001
X_3_ X_4_	182.31	1	182.31	29.87	< 0.0001
Residual	91.54	15	6.10		-
Lack of Fit	70.91	10	7.09	1.72	-
Pure Error	20.63	5	4.13	-	-
Corrected Total	12318.93	29	-	-	-
R-Squared		0.9926	Standard Deviation		2.47
Adjusted R^2^		0.9856	Coefficient of variation %		4.90
Adequate Precision		47.067	PRESS		438.15

**Figure 1:**
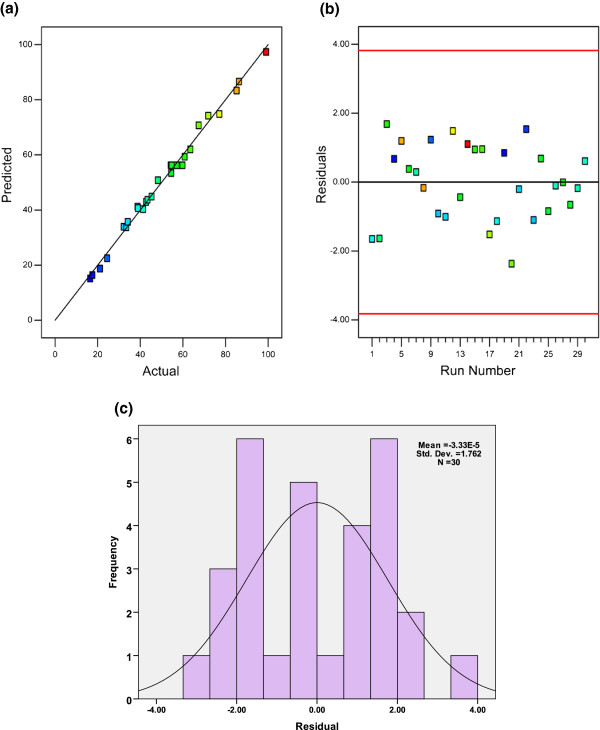
(a) Scatter plot of predicted photo degradation % value versus actual photo degradation % value (b) residual plot of model and (c) histogram of residuals with normal overlay.

R_d_^2^ provide a measure of how much variability in the observed response values can be explained by the experimental factors and their interactions. In this study, as obtained R_d_^2^ (0.9926) indicates that the model is capable of accounting for more than 99.26% of the variability in the responses. In addition, the R_Adj_^2^ (0.9856) is in reasonable agreement with (<0.20) with the R_Pred_^2^ (0.9644) which confirms the aptness of the model. Moreover, the adequate precision (47.067) shows remarkable signal (> > 4). These observations can be corroborated by regression plots. Further, Figure 1a shows the actual values versus predicted values of the photo degradation %, which indicated an excellent agreement between actual and predicted responses. A residual plot allowed visual assessment of the distance of each observation from the fitted line (Figure 1b). The residuals randomly scattered in a constant width band about the zero line. Figure 1 (c) shows the histogram of the residuals in allowed visual assessment of the assumption. As observed, the measurement errors in the response variable were normally distributed. This ensured model (quadratic) was suitable to navigate the design space and a satisfactory adjustment of the polynomial model to the experimental data.

### The quadratic expression model for the photo degradation

The quadratic model displayed in Eq. (3) expresses the relationship between responses of actual variables and the variables themselves.

(3) $$\begin{array}{ll} Y\;= & \;-616.18340\;+\;0.31631X_{1}\\ & +\;132.45247X_{2}-174.38646X_{3}\\ & -1.70016X_{4}–\;0.019661X_{1}X_{2}\\ & -\;0.026875\;X_{1}X_{3}+\;3.02083E\\ & -004\;X_{1}X_{4}–\;10.29688X_{2}X_{3}\\ & -20703\;X_{2}X_{4}–\;0,013750X_{3}X_{4}\\ & +\;1.67535E-004{X_{1}}^{2}\;–\;6.61621{X_{2}}^{2}\\ & -\;24.63750{X_{3}}^{2}\;+\;0.025781{X_{4}}^{2} \end{array}$$

Where X _1_, X _2_, X_3_ and X_4_ are demonstrated in Table 2. The positive sign in front of the terms indicates synergistic effect while negative sign indicates antagonistic effect. From the equation, the photo degradation % has been linear and quadratic effects by the four process variables (X _1_, X _2_, X_3_ and X_4_). The linear effects are irradiation time (X_1_), pH (*X*_2_), photo catalyst amount (X_3_), concentration of *o*-cresol (X_4_) and the second order effects are square of the variable (X_1_^2^, X_2_^2^, X_3_^2^ and X_4_^2^). In addition, the interactions effects of (X_1_X_2_, X_1_X_3_, X_1_X_4_, X_2_X_3_, X_2_X_4_) were observed in the model. The local optimums in terms of the actual variables can be determined by differentiating Eq. 3 for irradiation time (Eq. 4), pH (Eq. 5), amount of photo catalyst (Eq. 6), and *o*-cresol concentration (Eq. 7).

(4) $${\left[\partial Y/\partial X_{1}\right]}_{X_{2},X_{3},X_{4}}=0$$

(5) $${\left[\partial Y/\partial X_{2}\right]}_{X_{1},X_{3},X_{4}}=0$$

(6) $${\left[\partial Y/\partial X_{3}\right]}_{X_{1},X_{2},X_{4}}=0$$

(7) $${\left[\partial Y/\partial X_{4}\right]}_{X_{1},X_{2},X_{3}}=0$$

### Response surface 3D plots

**Figure 2:**
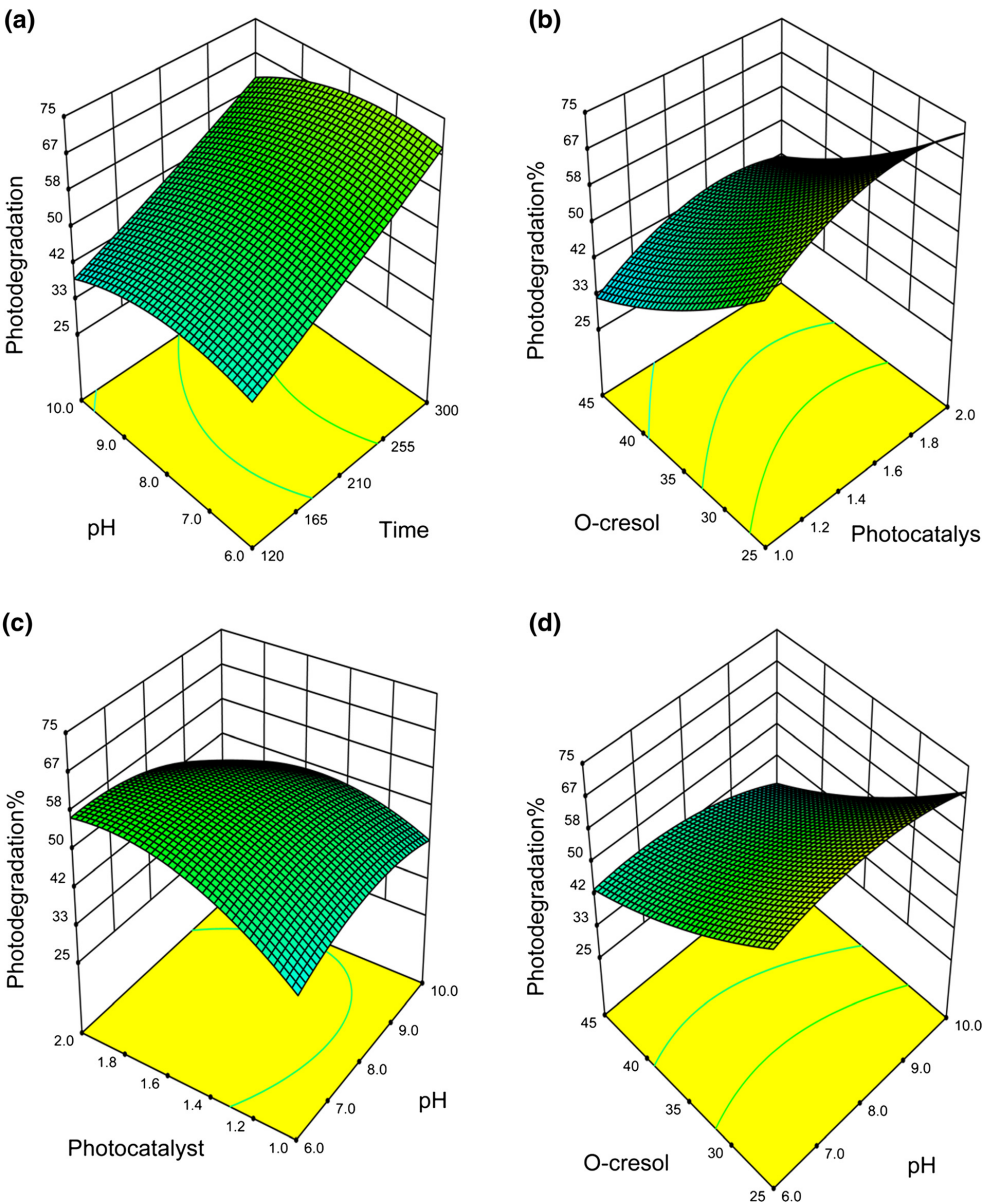
**Response surface 3D plots indicating the effect of interaction between process variables on photo degradation of *o*-cresol (a) Interaction between irradiation time and pH while holding the photo catalyst amount at 1.5 g/L and *o*-cresol concentration at 35 mg/L (b) Interaction between photo catalyst amount and *o*-cresol concentration while holding pH at 8.2 at end of 240 minutes of reaction time (c) Interaction between photo catalyst amount and pH while holding *o*-cresol concentration at 35 mg/L at end of 240 minutes of reaction time (d) Interaction between*o*-cresol concentration and pH while holding photo catalyst at1.5 g/L at end of 240 minutes of reaction time.**

Simulation is used when the real system cannot be engaged, because it may be inaccessible, dangerous, unacceptable, and expensive to perform. In photo degradation of o-cresol, the main limitations are expensive chemicals and instruments, time of experiments and numerous errors in the multiple experiments. Based on the validated model, the 3D plots presented the numerous predicted (simulated) responses with the four variables and one response (Table 2) of the photo degradation (Figure 2). As a preliminary study, the effect of pH, photo catalyst amount and o-cresol concentration on photo degradation was investigated during the irradiation time while two variables in each case held constant (e.g. Figure 2a). As observed, the photo degradation illustrated a peak at particular amount of pH, photo catalyst and *o*-cresol during the irradiation time. Therefore, a large numbers of experiments were simulated by end of 240 minutes of irradiation time while it was only one variable kept constant in each case (Figure b, c, d). Figure 2(b) shows the interaction between photo catalyst amount (1.0 – 2.0 g/L) and o-cresol concentration (25 – 45 mg/L) simultaneously with constant pH 8.2. As shown, the photo degradation % was decreased with increasing the *o*-cresol concentration for all the range of photo catalyst concentration. The reduction may be due to this reasons that *o*-cresol can be degraded directly by the generated holes (h^+^) over photo catalyst surface. In a high *o*-cresol concentrate solution, *o*-cresol molecules can compete with H_2_O to attract the h^+^ which is a limited agent [31]. On the other hand, the photo degradation % was increased with increasing photo catalyst amount up 1.6 g/L for all the concentration of o-cresol. This can be attributed to the fact that the increase in the effective surface area of the photo catalyst, which in turn leads to enhanced production of ^●^OH radicals. However, when the amount of photo catalyst was increased in excess of the optimum (1.6 g/L), the photo degradation % decreased. The decreased efficiency observed above the optimum photo catalyst loading may be attributed to the interception of light by the excess of photo catalyst particles in solution as known screen effect [8].

Figure 2(c) shows the interaction between pH (6-10) and photo catalyst amount (1.0 – 2.0 g/L) with constant o-cresol concentration 35 mg/L. As it is shown, the photo degradation % increased slightly with increasing pH from pH 7 to 9 in the range of photo catalyst amount. The increase in the photo degradation % may be due to increasing adsorption of *o*-cresol on the photo catalyst surface [32]. Moreover, It has been reported that, in slightly alkaline solution (pH=8), ^●^OH radicals are more easily generated by oxidizing the available OH^−^ on the photo catalyst surface [33]. Thus, generally, the photo degradation % is expected for becoming enhanced with increasing pH owing to the availability of ^●^OH radicals for the reaction. However, a decrease in photo degradation % was observed above the optimum. This can be attributed to the reduction for *o*-cresol adsorbed on the photo catalyst surface in the region of pH [31]. It should be noted as well that the radicals are rapidly scavenged in the presence of excess concentrations of OH^-^ and therefore would not have the opportunity to react with the substrates [33].

Figure 2(d) shows the interaction between pH (6-10) and o-cresol concentration (25 – 45 mg/L) with constant photo catalyst amount 1.5 g/L. As observed, the acceptable photo degradation % was obtained at 35 mg/L of o-cresol concentration. Any increase in the concentration resulted in diminishing photo degradation %. The decrease in photo degradation % may be due to the reason that the *o*-cresol concentration increased while the active sites of photo catalyst remained constant [34]. As a result, the optimum photo degradation was 60% (average) in the condition (pH 8.2, photo catalyst amount 1.6 g/L and o-cresol concentration 35 mg/L at 240 minutes of irradiation time). The optimum was validated by performing the similar experimental methodology [20]. As observed, the experimental values were reasonably close to the simulated values that indicated the high validity and adequacy of the model.

## Conclusion

The optimization and modeling of *o*-cresol photo degradation was studied by RSM. The experiments were designed with four effective factors including irradiation time, pH, photo catalyst’s amount and *o*-cresol concentration by the CCD. The CCD considered 30 runs to obtain actual responses. To suggest a model for the photo degradation process, the responses were fitted with a quadratic model. The ANOVA confirmed the high validity of the model by using excellent evidences such as high F-value (143.12), very low P-value (<0.0001), non-significant lack of fit, the R^2^ (0.99), and the adequate precision (47.067). The results of simulated 3D plots for the photo degradation (60%) were agreed with experimental results (63%). This study indicates the success of RSM to simulate the optimum condition of *o*-cresol photo degradation % under visible-light by Mn-doped ZnO nanoparticles as photo catalyst.

## Competing interests

The authors declare that they have no competing interests.

## Authors' contributions

Yadollah Abdollahi (AB, JY, MT); Azmi Zakaria(FG); Abdul Halim Abdullah (FG); Hamid Reza Fard Masoumi (MT); Hossein Jahangirian (JY); Kamyar Shameli (JY); Majid Rezayi (JY); Santo Banerjee (MT); Tahereh Abdollahi (JY, MT). AB carried out the catalyst design and ligand screening studies. JY carried out the synthesis, purification and characterization of the compounds. MT carried out the computational experiments. FG conceived of the study, and participated in its design and coordination and helped to draft the manuscript. All authors read and approved the final manuscript.

## References

[B1] Jo W Shin M Visible-light-activated photo catalysis of malodorous dimethyl disulphide using nitrogen-enhanced TiO_2_ Environ Technol 2010 31 575 584 10.1080/09593330903536121 20480832

[B2] Ngouyap Mouamfon MV Li W Lu S Qiu Z Chen N Lin K Photo degradation of sulphamethoxazole under UV-light irradiation at 254 nm Environ Technol 2010 31 489 494 10.1080/09593330903514854 20480824

[B3] Poulios I Aetopoulou I Photo catalytic degradation of the textile dye reactive orange 16 in the presence of TiO_2_ suspensions. Environ Technol 1999 20 479 487 10.1080/09593332008616843

[B4] Tang W Zhang Z An H Quintana M Torres D TiO_2_/UV photo degradation of azo dyes in aqueous solutions. Environ Technol 1997 18 1 12

[B5] Glaze W Drinking-water treatment with ozone Environ Sci Technol 1987 21 224 230 10.1021/es00157a001 22185096

[B6] Litter MI Heterogeneous photo catalysis: Transition metal ions in photo catalytic systems Appl Catalo Environ 1999 23 89 114 10.1016/S0926-3373(99)00069-7

[B7] Peiró AM Ayllón JA Peral J Doménech X TIO_2_-photocatalyzed degradation of phenol and ortho-substituted phenolic compounds. Appl Catal Environ 2001 30 359 373 10.1016/S0926-3373(00)00248-4

[B8] Pardeshi SK Patil AB A simple route for photo catalytic degradation of phenol in aqueous zinc oxide suspension using solar energy Solar Energy 2008 82 700 705 10.1016/j.solener.2008.02.007

[B9] Dindar B Içli S Unusual photo reactivity of zinc oxide irradiated by concentrated sunlight Journal of Photochemistry and Photobiology A: Chemistry 2001 140 263 268 10.1016/S1010-6030(01)00414-2

[B10] Sakthivel S Neppolian B Shankar M Arabindoo B Palanichamy M Murugesan V Solar photo catalytic degradation of azo dye: comparison of photo catalytic efficiency of ZnO and TiO_2_. Solar Energy Materials and Solar Cells 2003 77 65 82 10.1016/S0927-0248(02)00255-6

[B11] Abdollahi Y Abdullah AH Zainal Z Yusof NA Photo degradation of p-cresol by Zinc Oxide under Visible Light International Journal of Applied Science and Technology 2011 1 99 105 10.3390/ijms13010302 PMC3269687 22312253

[B12] Abdollahi Y Abdullah AH Zainal Z Yusof NA Synthesis and characterization of Manganese doped ZnO nanoparticles International Journal of Basic & Applied Sciences 2011 11 62 69

[B13] Abdel-Fattah YR Saeed HM Gohar YM El-Baz MA Improved production of Pseudomonas aeruginosa uricase by optimization of process parameters through statistical experimental designs Process Biochem 2005 40 1707 1714 10.1016/j.procbio.2004.06.048

[B14] Bas D Boyacı IH Modeling and optimization I: Usability of response surface methodology J Food Eng 2007 78 836 845 10.1016/j.jfoodeng.2005.11.024

[B15] Lin Y Ferronato C Deng N Wu F Chovelon J-M Photo catalytic degradation of methylparaben by TiO_2_: Multivariable experimental design and mechanism. Appl Catal Environ 2009 88 32 41 10.1016/j.apcatb.2008.09.026

[B16] Sharma A Rao P Mathur RP Ameta SC Photo catalytic reactions of xylidine ponceau on semiconducting zinc oxide powder Journal of Photochemistry and Photobiology A: Chemistry 1995 86 197 200 10.1016/1010-6030(94)03933-L

[B17] Daneshvar Aber S Seyed Dorraji M Khataee A Rasoulifard M Photo catalytic degradation of the insecticide diazinon in the presence of prepared nanocrystalline ZnO powders under irradiation of UV-C light Sep Purif Technol 2007 58 91 98 10.1016/j.seppur.2007.07.016

[B18] Kansal SK Singh M Sud D Studies on TiO_2_/ZnO photocatalysed degradation of lignin J Hazard Mater 2008 153 412 417 10.1016/j.jhazmat.2007.08.091 17936502

[B19] Akyol A Yatmaz HC Bayramoglu M Photo catalytic decolorization of Remazol Red RR in aqueous ZnO suspensions Appl Catal Environ 2004 54 19 24 10.1016/j.apcatb.2004.05.021

[B20] Abdollahi Y Abdullah AH Zainal Z Yusof NA Photo degradation of m-cresol by Zinc Oxide under Visible-light Irradiation Int J Chem 2011 3 31 43

[B21] Lin Y Ferronato C Deng N Wu F Chovelon JM Photo catalytic degradation of methylparaben by TiO_2_: Multivariable experimental design and mechanism Appl Catal Environ 2009 88 32 41 10.1016/j.apcatb.2008.09.026

[B22] Sin JC Lam SM Mohamed AR Optimizing photo catalytic degradation of phenol by TiO 2/GAC using response surface methodology Korean Journal of Chemical Engineering 2011 28 84 92 10.1007/s11814-010-0318-0

[B23] Cho IH Zoh KD Photo catalytic degradation of azo dye (Reactive Red 120) in TiO_2_/UV system: Optimization and modeling using a response surface methodology (RSM) based on the central composite design. Dyes Pigments 2007 75 533 543 10.1016/j.dyepig.2006.06.041

[B24] YuGui T LianBin Y Jun P YaoMing W Bin T Removal of Pb (II) from aqueous solution on chitosan/TiO_2_ hybrid film. J Hazard Mater 2009 161 718 722 10.1016/j.jhazmat.2008.04.012 18495341

[B25] Yeber MC Soto C Riveros R Navarrete J Vidal G Optimization by factorial design of copper (II) and toxicity removal using a photo catalytic process with TiO_2_ as semiconductor. Chem Eng J 2009 152 14 19 10.1016/j.cej.2009.03.021

[B26] Sakkas V Calza P Islam MA Medana C Baiocchi C Panagiotou K Albanis T TiO_2_/H_2_O_2_ mediated photo catalytic transformation of UV filter 4-methylbenzylidene camphor (4-MBC) in aqueous phase: Statistical optimization and photoproduct analysis. Appl Catal Environ 2009 90 526 534 10.1016/j.apcatb.2009.04.013

[B27] Montgomery DC Design and analysis of experiments 2008 United States, New York: John Wiley & Sons Inc

[B28] Chen CS Liu KJ Lou YH Shieh CJ Optimization of kojic acid monolaurate synthesis with lipase PS from Pseudomonas cepacia J Sci Food Agric 2002 82 601 605 10.1002/jsfa.1083

[B29] Abdollahi Y Abdullah A Gaya U Zainal Z Yusof N Enhanced photo degradation of o-cresol in aqueous Mn (1%)-doped ZnO suspensions Environ Technol 2011 33 1 7 10.1080/09593330.2011.618931 22856288

[B30] Liu HL Chiou YR Optimal decolorization efficiency of Reactive Red 239 by UV/TiO_2_ photo catalytic process coupled with response surface methodology. Chem Eng J 2005 112 173 179 10.1016/j.cej.2005.07.012

[B31] Abdollahi Y Abdullah AH Zakaria A Zainal Z Masoumi HRF Yusof NA Photo degradation of p-cresol in Aqueous Mn (1%)-Doped ZnO Suspensions Journal of Advanced Oxidation Technologies 2012 15 146 152

[B32] Konstantinou IK Albanis TA TiO_2_-assisted photo catalytic degradation of azo dyes in aqueous solution: kinetic and mechanistic investigations: A review Appl Catal Environ 2004 49 1 14 10.1016/j.apcatb.2003.11.010

[B33] Davis A Huang C Removal of phenols from water by a photo catalytic oxidation process Water Sci Technol 1989 21 455 464

[B34] Lathasree S Rao A SivaSankar B Sadasivam V Rengaraj K Heterogeneous photo catalytic mineralization of phenols in aqueous solutions Journal of Molecular Catalysis A: Chemical 2004 223 101 105 10.1016/j.molcata.2003.08.032

